# RETRACTION: miR‐664a‐3p functions as an oncogene by targeting Hippo pathway in the development of gastric cancer

**DOI:** 10.1111/cpr.13681

**Published:** 2024-05-28

## Abstract

**RETRACTION:** L. Wang, B. Li, L. Zhang, Q. Li, Z. He, X. Zhang, X. Huang, Z. Xu, Y. Xia, Q. Zhang, Q. Li, J. Xu, G. Sun, Z. Xu, “miR‐664a‐3p functions as an oncogene by targeting Hippo pathway in the development of gastric cancer,” *Cell Proliferation* 52, no. 3 (2019): e12567, https://doi.org/10.1111/cpr.12567.

The above article, published online on 18 March 2019, in Wiley Online Library (wileyonlinelibrary.com), has been retracted by agreement between the journal Editor‐in‐Chief, Qi Zhou, and John Wiley and Sons Ltd. Following publication, concerns were raised by third parties regarding suspected duplication of figures 2c, 2d, 4g, 4h and 5a. The authors explained that the duplicates were a result of negligence during data storage. Due to the extent and nature of the mistakes made, the editors have lost confidence in the results and conclusions of this study.

**RETRACTION:** L. Wang, B. Li, L. Zhang, Q. Li, Z. He, X. Zhang, X. Huang, Z. Xu, Y. Xia, Q. Zhang, Q. Li, J. Xu, G. Sun, Z. Xu, “miR‐664a‐3p functions as an oncogene by targeting Hippo pathway in the development of gastric cancer,” *Cell Proliferation* 52, no. 3 (2019): e12567, https://doi.org/10.1111/cpr.12567.

The above article, published online on 18 March 2019, in Wiley Online Library (wileyonlinelibrary.com), has been retracted by agreement between the journal Editor‐in‐Chief, Qi Zhou, and John Wiley and Sons Ltd. Following publication, concerns were raised by third parties regarding suspected duplication of figures 2c, 2d, 4g, 4h and 5a. The authors explained that the duplicates were a result of negligence during data storage. Due to the extent and nature of the mistakes made, the editors have lost confidence in the results and conclusions of this study.

